# Ethyl 4-(5-bromo-1*H*-indol-3-yl)-2,6,6-trimethyl-5-oxo-1,4,5,6,7,8-hexa­hydro­quinoline-3-carboxyl­ate

**DOI:** 10.1107/S1600536812046909

**Published:** 2012-11-24

**Authors:** Miyase Gözde Gündüz, Ray J. Butcher, Sema Öztürk Yildirim, Ahmed El-Khouly, Cihat Şafak, Rahime Şimşek

**Affiliations:** a Hacettepe University, Faculty of Pharmacy, Department of Pharmaceutical Chemistry, 06100 Sihhiye-Ankara, Turkey; b Department of Chemistry, Howard University, 525 College Street NW, Washington, DC 20059, USA; c Department of Physics, Faculty of Sciences, Erciyes University, 38039 Kayseri, Turkey

## Abstract

The title compound, C_23_H_25_BrN_2_O_3_, crystallizes with two independent mol­ecules in the asymmetric unit (*Z*′ = 2) which differ in the twist of the 5-bromo-1*H*-indole ring with respect to the plane of the 4-methyl-1,4,5,6,7,8-hexa­hydro­quinoline ring [dihedral angles of 78.55 (9) and 89.70 (8)° in molecules *A* and *B*, respectively]. The indole ring is planar in both molecules [maximum deviations = 0.021 (3) and −0.020 (3) Å for the N atom] while the cyclo­hexene ring has adopts a sofa conformation. In the crystal, mol­ecules are linked by pairs of N—H⋯O hydrogen bonds, forming dimers with *R*_1_^2^(6) ring motifs. These dimers are connected by N—H⋯O hydrogen bonds, generating chains along [110]. A C—H⋯O contact occurs between the independent mol­ecules.

## Related literature

For biological properties of 1,4-dihydro­pyridines, see: Triggle, (2003 ▶); Şafak & Şimşek (2006 ▶). For the introduction of nifedipine into clinical use, see: Gordeev *et al.* (1998 ▶). For a description of the Cambridge Structural Database, see: Allen, (2002 ▶). For geometric analysis, see: Cremer & Pople (1975 ▶). For hydrogen-bond motifs, see: Bernstein *et al.* (1995 ▶); Etter *et al.* (1990 ▶). For similar structures, see: El-Khouly *et al.* (2012 ▶); Öztürk Yildirim *et al.* (2012 ▶).
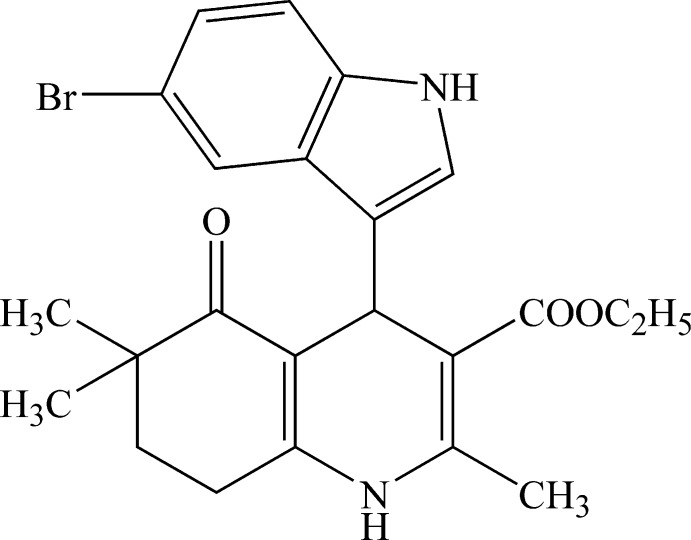


## Experimental

### 

#### Crystal data

C_23_H_25_BrN_2_O_3_
*M* *_r_* = 457.36
Monoclinic, 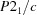

*a* = 14.0044 (6) Å
*b* = 16.8802 (5) Å
*c* = 18.8341 (7) Å
β = 105.582 (4)°
*V* = 4288.7 (3) Å^3^
*Z* = 8
Cu *K*α radiation
μ = 2.83 mm^−1^
*T* = 123 K
0.83 × 0.69 × 0.48 mm

#### Data collection

Agilent Xcalibur (Ruby, Gemini) diffractometer
Absorption correction: analytical [(Clark & Reid, 1995 ▶) in *CrysAlis RED* (Agilent (2011 ▶)] *T*_min_ = 0.270, *T*_max_ = 0.514
17300 measured reflections
8621 independent reflections
7227 reflections with *I* > 2σ(*I*)
*R*_int_ = 0.038

#### Refinement

*R*[*F* ^2^ > 2σ(*F* ^2^)] = 0.058
*wR*(*F* ^2^) = 0.159
*S* = 1.02
8621 reflections
531 parameters
H-atom parameters constrained
Δρ_max_ = 1.31 e Å^−3^
Δρ_min_ = −1.01 e Å^−3^

### 

Data collection: *CrysAlis PRO* (Agilent, 2011 ▶); cell refinement: *CrysAlis PRO*; data reduction: *CrysAlis PRO*; program(s) used to solve structure: *SHELXS97* (Sheldrick, 2008 ▶); program(s) used to refine structure: *SHELXL97* (Sheldrick, 2008 ▶); molecular graphics: *SHELXTL* (Sheldrick, 2008 ▶); software used to prepare material for publication: *SHELXTL*.

## Supplementary Material

Crystal structure: contains datablock(s) I, global. DOI: 10.1107/S1600536812046909/hg5269sup1.cif

Structure factors: contains datablock(s) I. DOI: 10.1107/S1600536812046909/hg5269Isup2.hkl

Supplementary material file. DOI: 10.1107/S1600536812046909/hg5269Isup3.cml

Additional supplementary materials:  crystallographic information; 3D view; checkCIF report

## Figures and Tables

**Table 1:** Hydrogen-bond geometry (Å, °)

*D*—H⋯*A*	*D*—H	H⋯*A*	*D*⋯*A*	*D*—H⋯*A*
C2*A*—H2*AB*⋯O1*B*	0.99	2.51	3.296 (4)	136
N1*A*—H1*AA*⋯O1*B*	0.88	2.01	2.870 (3)	165
N2*A*—H2*AC*⋯O2*B* ^i^	0.88	1.94	2.807 (3)	167
N1*B*—H1*BA*⋯O1*A* ^ii^	0.88	1.97	2.845 (3)	173
N2*B*—H2*BC*⋯O2*A* ^iii^	0.88	2.05	2.889 (3)	159

Symmetry codes: (i) 
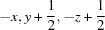
; (ii) 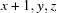; (iii) 
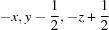
.

**Fig. 1.**
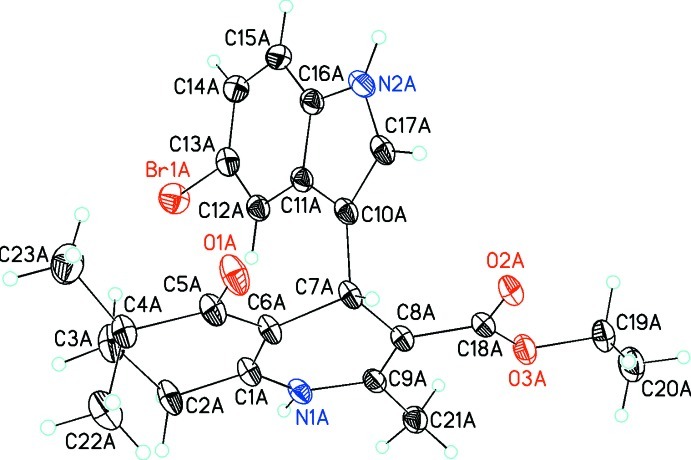
Molecule A of the title compound showing the conformation of the 5-bromo-1H-indole ring with respect to the hexahydro-quinoline ring plane. Displacement ellipsoids are drawn at the 30% probability level and H atoms are shown as small spheres of arbitrary radius.

**Fig. 2.**
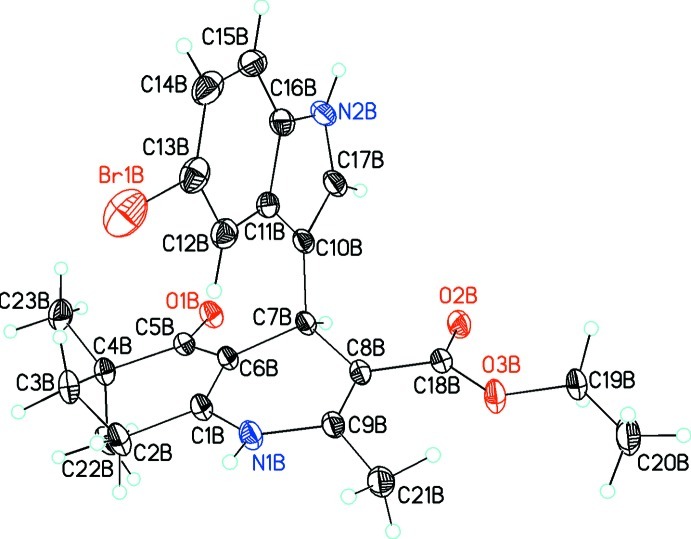
Molecule B of the title compound showing the conformation of the 5-bromo-1H-indole ring with respect to the hexahydro-quinoline ring plane. Displacement ellipsoids are drawn at the 30% probability level and H atoms are shown as small spheres of arbitrary radius.

**Fig. 3.**
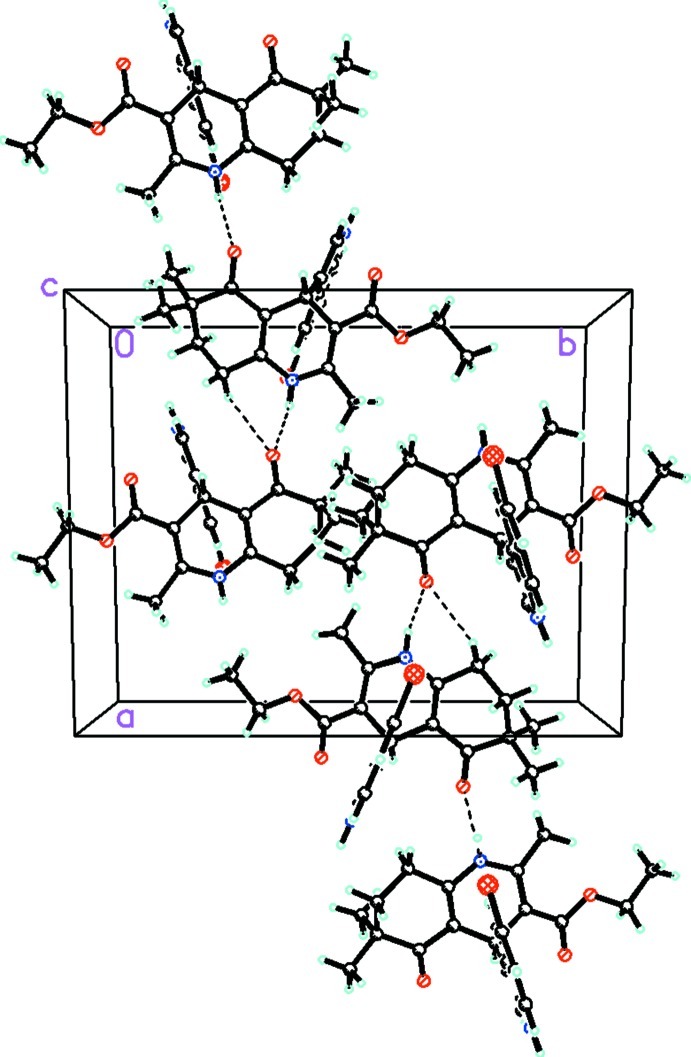
The crystal packing of the title compound, viewing along the b axis. Dashed lines show the intermolecular hydrogen bonding interactions.

## supplementary crystallographic information

### Comment

1,4-Dihydropyridines (DHP), of which nifedipine is the prototype for this group, are one of the known classes of calcium antagonists, which reversibly block Ca^2+^ influx through *L*-type calcium channels and are frequently used for the treatment of cardiovascular diseases like angina, hypertension and supraventricular tachycardia (Triggle, 2003; Şafak & Şimşek, 2006).

Since the introduction of nifedipine into clinical use, many modifications have been made such as fusing one of the carbonyl groups into the ring system or substitution of the phenyl ring with heteroaromatic rings in order to elucidate the SARs (severe acute respiratory syndrome) and to enhance calcium-modulating effects (Gordeev *et al.*, 1998). Following on from these results, we synthesized the title compound and determined its structure.

In the title compound (I), (Fig. 1, Fig. 2), the 5-bromo-1*H*-indole ring is almost planar in both of the molecules with a maximum deviation from the mean plane of 0.021 (3) Å for atom N2A and -0.020 (3) Å for atom N2B. The cyclohexene rings adopt a sofa conformation and are puckered with puckering parameters in molecule A and B (Cremer & Pople, 1975) of Q_T_ = 0.472 (4) Å, θ = 120.7 (5) °, φ = 295.1 (5) ° and Q_T_ = 0.475 (4) Å, θ = 62.7 (4) °, φ = 120.5 (4) °, respectively. The values of the bond lengths (Allen, 2002) and angles in (I) are in normal ranges and are comparable with those of related structures (El-Khouly *et al.*, 2012; Öztürk Yildirim *et al.*, 2012).

In the crystal, molecules are linked by pairs of intermolecular N—H···O hydrogen bonds, forming dimers with *R*_1_^2^(6) ring motifs (Bernstein *et al.*, 1995; Etter *et al.*, 1990), and these dimers are connected by N—H···O hydrogen bonds, generating one-dimensional chains along [110] (Table 1, Fig. 3).

### Experimental

The title compound was prepared by refluxing 4,4-dimethyl-1,3-cyclohexanedione (0.001 mol), ethyl acetoacetate (0.001 mol), 6-bromoindole-2-carbaldehyde (0.001 mol) and ammonium acetate (0.005 mol) in methanol for 8 h. Crystals were grown by slow evaporation of a methanol solution. The structure of the compound was elucidated by IR, ^1^H-NMR and elemental analysis. *M*.p. 487 K; IR (cm^-1^): 3297, 3087, 2983, 1688; ^1^H-NMR δ (p.p.m.) 0.9–1.0 (6H; s; 2xCH_3_), 1.1 (3H; t; CH_2_CH_3_), 1.6–1.8 (6H; m; quinoline H7,8), 2.2 (3H; s; CH_3_), 4.0 (2H; m; CH_2_CH_3_), 5.2 (1H; s; quinoline H4), 6.9 (1H; s; indole H3), 7.1–7.7 (3H; m; aromatic), 9.2 (1H; s; quinoline NH), 10.8 (1H; s; indole NH). Anal. for C_23_H_25_BrN_2_O_3_ calculated: C, 60.40; H, 5.51; N, 6.13; found: C, 59.89; H, 5.86; N, 6.36. The compound demonstrated calcium channel blocker activity in isolated rat ileum and lamb carotid artery.

### Refinement

All H atoms were placed in idealized positions (C—H = 0.95–0.99 Å and N—H = 0.88 Å) and refined as riding atoms with *U*_iso_(H) = 1.2*U*_eq_(C or N) or 1.5 *U*_eq_(C_methyl_). A rotating-group model was applied for the methyl groups. The highest residual electron density peak and the deepest hole are located at 0.86 and 0.82 Å from Br1B, respectively. Forty three outliers,(1 0 0), (-3 6 2), (2 4 0(, (-1 2 7), (1 3 6), (-4 6 2), (1 4 1), (3 1 3), (4 5 1), (-1 0 4), (-11 5 5), (-2 0 12), (-8 6 10), (-3 1 8), (-4 2 4), (3 1 2), (0 5 3), (6 2 1), (-2 1 8), (4 8 9), (-3 3 9), (-7 0 12), (-3 2 8), (-9 3 5), (-8 7 6), (-1 1 1), (-1 1 10), (-4 4 1), (-4 2 5), (-9 9 20), (-3 9 1), (-6 2 3), (-2 0 6), (-9 4 5), (-1 7 3), (3 1 5), (2 4 3), (2 4 4), (-11 3 5), (0 0 6), (0 1 10), (-5 3 2) and (1 8 3), were omitted in the final refinement.

### Crystal data

**Table:**

C_23_H_25_BrN_2_O_3_	*F*(000) = 1888
*M**_r_* = 457.36	*D*_x_ = 1.417 Mg m^−^^3^
Monoclinic, *P*2_1_/*c*	Cu *K*α radiation, λ = 1.54184 Å
Hall symbol: -P 2ybc	Cell parameters from 6306 reflections
*a* = 14.0044 (6) Å	θ = 3.3–75.5°
*b* = 16.8802 (5) Å	µ = 2.83 mm^−^^1^
*c* = 18.8341 (7) Å	*T* = 123 K
β = 105.582 (4)°	Irregular, colorless
*V* = 4288.7 (3) Å^3^	0.83 × 0.69 × 0.48 mm
*Z* = 8	

### Data collection

**Table:**

Agilent Xcalibur (Ruby, Gemini) diffractometer	8621 independent reflections
Radiation source: Enhance (Cu) X-ray Source	7227 reflections with *I* > 2σ(*I*)
Graphite monochromator	*R*_int_ = 0.038
Detector resolution: 10.5081 pixels mm^-1^	θ_max_ = 75.7°, θ_min_ = 3.6°
ω scans	*h* = −17→16
Absorption correction: analytical [(Clark & Reid, 1995) implemented in *CrysAlis RED* (Agilent (2011)]	*k* = −18→21
*T*_min_ = 0.270, *T*_max_ = 0.514	*l* = −23→22
17300 measured reflections	

### Refinement

**Table:**

Refinement on *F*^2^	Primary atom site location: structure-invariant direct methods
Least-squares matrix: full	Secondary atom site location: difference Fourier map
*R*[*F*^2^ > 2σ(*F*^2^)] = 0.058	Hydrogen site location: inferred from neighbouring sites
*wR*(*F*^2^) = 0.159	H-atom parameters constrained
*S* = 1.02	*w* = 1/[σ^2^(*F*_o_^2^) + (0.078*P*)^2^ + 4.2146*P*] where *P* = (*F*_o_^2^ + 2*F*_c_^2^)/3
8621 reflections	(Δ/σ)_max_ = 0.001
531 parameters	Δρ_max_ = 1.31 e Å^−^^3^
0 restraints	Δρ_min_ = −1.01 e Å^−^^3^

### Special details

**Table:**

Experimental. The crystals were very fragile. On cutting the crystals shattered so the smallest viable crystal was selected and an incident collimator of 1.0 mm was used.Analytical numeric absorption correction using a multifaceted crystal model (Clark & Reid, 1995).
Geometry. All e.s.d.'s (except the e.s.d. in the dihedral angle between two l.s. planes) are estimated using the full covariance matrix. The cell e.s.d.'s are taken into account individually in the estimation of e.s.d.'s in distances, angles and torsion angles; correlations between e.s.d.'s in cell parameters are only used when they are defined by crystal symmetry. An approximate (isotropic) treatment of cell e.s.d.'s is used for estimating e.s.d.'s involving l.s. planes.
Refinement. Refinement of *F*^2^ against ALL reflections. The weighted *R*-factor *wR* and goodness of fit *S* are based on *F*^2^, conventional *R*-factors *R* are based on *F*, with *F* set to zero for negative *F*^2^. The threshold expression of *F*^2^ > σ(*F*^2^) is used only for calculating *R*-factors(gt) *etc*. and is not relevant to the choice of reflections for refinement. *R*-factors based on *F*^2^ are statistically about twice as large as those based on *F*, and *R*- factors based on ALL data will be even larger.

### Fractional atomic coordinates and isotropic or equivalent isotropic displacement parameters (Å^2^)

**Table:**

	*x*	*y*	*z*	*U*_iso_*/*U*_eq_	
Br1A	0.13692 (3)	0.37770 (3)	0.09007 (2)	0.05924 (14)	
O1A	−0.15665 (18)	0.27492 (13)	0.32627 (17)	0.0493 (6)	
O2A	−0.09319 (17)	0.55281 (13)	0.41215 (13)	0.0420 (5)	
O3A	0.05958 (19)	0.60408 (13)	0.43578 (15)	0.0469 (5)	
N1A	0.15943 (18)	0.38895 (14)	0.36909 (15)	0.0355 (5)	
H1AA	0.2210	0.3836	0.3668	0.043*	
N2A	−0.22154 (19)	0.49209 (15)	0.17494 (17)	0.0400 (6)	
H2AC	−0.2782	0.5135	0.1505	0.048*	
C1A	0.0954 (2)	0.32637 (17)	0.34815 (18)	0.0365 (6)	
C2A	0.1409 (2)	0.24903 (18)	0.3337 (2)	0.0473 (8)	
H2AA	0.1666	0.2200	0.3807	0.057*	
H2AB	0.1970	0.2595	0.3124	0.057*	
C3A	0.0613 (3)	0.1980 (2)	0.2793 (3)	0.0539 (9)	
H3AA	0.0440	0.2235	0.2301	0.065*	
H3AB	0.0896	0.1452	0.2743	0.065*	
C4A	−0.0320 (2)	0.18776 (18)	0.3045 (2)	0.0411 (7)	
C5A	−0.0703 (2)	0.26800 (17)	0.3243 (2)	0.0396 (7)	
C6A	−0.0030 (2)	0.33535 (16)	0.34118 (18)	0.0359 (6)	
C7A	−0.0463 (2)	0.41683 (16)	0.34580 (18)	0.0331 (6)	
H7AA	−0.0962	0.4119	0.3749	0.040*	
C8A	0.0331 (2)	0.47467 (16)	0.38612 (17)	0.0335 (6)	
C9A	0.1307 (2)	0.46069 (17)	0.39384 (17)	0.0348 (6)	
C10A	−0.1001 (2)	0.44528 (16)	0.26938 (17)	0.0326 (6)	
C11A	−0.0690 (2)	0.43850 (16)	0.20286 (17)	0.0331 (6)	
C12A	0.0175 (2)	0.41221 (17)	0.18585 (18)	0.0366 (6)	
H12A	0.0727	0.3937	0.2234	0.044*	
C13A	0.0202 (3)	0.41393 (19)	0.1136 (2)	0.0424 (7)	
C14A	−0.0594 (3)	0.4412 (2)	0.0561 (2)	0.0476 (8)	
H14A	−0.0549	0.4403	0.0067	0.057*	
C15A	−0.1440 (3)	0.4691 (2)	0.0719 (2)	0.0461 (7)	
H15A	−0.1980	0.4889	0.0340	0.055*	
C16A	−0.1481 (2)	0.46742 (17)	0.14475 (19)	0.0379 (6)	
C17A	−0.1929 (2)	0.47820 (16)	0.24887 (19)	0.0358 (6)	
H17A	−0.2320	0.4898	0.2818	0.043*	
C18A	−0.0062 (2)	0.54629 (17)	0.41275 (17)	0.0360 (6)	
C19A	0.0234 (3)	0.6765 (2)	0.4605 (2)	0.0510 (8)	
H19A	−0.0025	0.6659	0.5036	0.061*	
H19B	−0.0306	0.6994	0.4207	0.061*	
C20A	0.1085 (4)	0.7317 (2)	0.4809 (3)	0.0671 (12)	
H20A	0.0874	0.7816	0.4985	0.101*	
H20B	0.1330	0.7420	0.4376	0.101*	
H20C	0.1616	0.7080	0.5200	0.101*	
C21A	0.2166 (2)	0.5128 (2)	0.4294 (2)	0.0444 (7)	
H21A	0.2141	0.5260	0.4796	0.067*	
H21B	0.2133	0.5616	0.4007	0.067*	
H21C	0.2787	0.4849	0.4314	0.067*	
C22A	−0.0118 (3)	0.1373 (2)	0.3741 (3)	0.0598 (10)	
H22A	0.0372	0.1639	0.4140	0.090*	
H22B	0.0139	0.0855	0.3645	0.090*	
H22C	−0.0735	0.1301	0.3885	0.090*	
C23A	−0.1124 (3)	0.1468 (3)	0.2437 (3)	0.0639 (11)	
H23A	−0.0875	0.0956	0.2317	0.096*	
H23B	−0.1294	0.1803	0.1996	0.096*	
H23C	−0.1716	0.1383	0.2611	0.096*	
Br1B	0.63774 (4)	0.23757 (4)	0.06648 (3)	0.07158 (17)	
O1B	0.34556 (15)	0.34914 (12)	0.34170 (13)	0.0369 (4)	
O2B	0.40595 (16)	0.06588 (13)	0.38178 (14)	0.0421 (5)	
O3B	0.55806 (17)	0.01606 (12)	0.39880 (14)	0.0423 (5)	
N1B	0.65221 (18)	0.23898 (14)	0.34324 (16)	0.0354 (5)	
H1BA	0.7118	0.2459	0.3371	0.042*	
N2B	0.2680 (2)	0.14747 (15)	0.14840 (16)	0.0416 (6)	
H2BC	0.2096	0.1298	0.1233	0.050*	
C1B	0.5887 (2)	0.30229 (16)	0.33371 (16)	0.0314 (5)	
C2B	0.6358 (2)	0.38185 (17)	0.3312 (2)	0.0415 (7)	
H2BA	0.6851	0.3781	0.3023	0.050*	
H2BB	0.6708	0.3988	0.3818	0.050*	
C3B	0.5566 (3)	0.44283 (18)	0.2960 (2)	0.0419 (7)	
H3BA	0.5316	0.4313	0.2427	0.050*	
H3BB	0.5871	0.4962	0.3013	0.050*	
C4B	0.4689 (2)	0.44316 (16)	0.33058 (17)	0.0351 (6)	
C5B	0.4289 (2)	0.35879 (16)	0.33301 (15)	0.0298 (5)	
C6B	0.4914 (2)	0.29147 (15)	0.32859 (15)	0.0291 (5)	
C7B	0.44794 (19)	0.20870 (15)	0.32303 (15)	0.0280 (5)	
H7BA	0.3990	0.2077	0.3531	0.034*	
C8B	0.5283 (2)	0.14830 (16)	0.35610 (16)	0.0304 (5)	
C9B	0.6255 (2)	0.16410 (16)	0.36236 (17)	0.0327 (6)	
C10B	0.3929 (2)	0.18810 (15)	0.24485 (16)	0.0298 (5)	
C11B	0.4259 (2)	0.19214 (15)	0.17849 (16)	0.0321 (6)	
C12B	0.5150 (2)	0.21294 (17)	0.16260 (18)	0.0377 (6)	
H12B	0.5711	0.2292	0.2005	0.045*	
C13B	0.5185 (3)	0.2090 (2)	0.09021 (19)	0.0448 (7)	
C14B	0.4370 (3)	0.1861 (2)	0.03215 (19)	0.0516 (9)	
H14B	0.4424	0.1855	−0.0170	0.062*	
C15B	0.3497 (3)	0.16487 (18)	0.04660 (18)	0.0475 (8)	
H15B	0.2941	0.1493	0.0079	0.057*	
C16B	0.3447 (2)	0.16667 (16)	0.11969 (18)	0.0394 (7)	
C17B	0.2974 (2)	0.16038 (16)	0.22313 (19)	0.0376 (6)	
H17B	0.2570	0.1513	0.2555	0.045*	
C18B	0.4912 (2)	0.07421 (16)	0.37957 (16)	0.0321 (6)	
C19B	0.5237 (3)	−0.05911 (17)	0.42088 (19)	0.0424 (7)	
H19C	0.4734	−0.0832	0.3792	0.051*	
H19D	0.4938	−0.0512	0.4624	0.051*	
C20B	0.6131 (3)	−0.1108 (2)	0.4437 (3)	0.0647 (12)	
H20D	0.5960	−0.1597	0.4655	0.097*	
H20E	0.6655	−0.0829	0.4801	0.097*	
H20F	0.6364	−0.1238	0.4005	0.097*	
C21B	0.7139 (2)	0.11148 (19)	0.3907 (2)	0.0474 (8)	
H21D	0.6951	0.0563	0.3777	0.071*	
H21E	0.7375	0.1165	0.4444	0.071*	
H21F	0.7667	0.1272	0.3684	0.071*	
C22B	0.5021 (3)	0.47315 (19)	0.4104 (2)	0.0448 (7)	
H22D	0.5232	0.5285	0.4108	0.067*	
H22E	0.5576	0.4409	0.4385	0.067*	
H22F	0.4467	0.4692	0.4328	0.067*	
C23B	0.3868 (3)	0.49594 (19)	0.2847 (2)	0.0476 (8)	
H23G	0.4127	0.5496	0.2825	0.071*	
H23D	0.3317	0.4978	0.3075	0.071*	
H23E	0.3635	0.4744	0.2348	0.071*	

### Atomic displacement parameters (Å^2^)

**Table:**

	*U*^11^	*U*^22^	*U*^33^	*U*^12^	*U*^13^	*U*^23^
Br1A	0.0535 (2)	0.0716 (3)	0.0595 (2)	0.01058 (19)	0.02695 (19)	0.00035 (19)
O1A	0.0372 (12)	0.0293 (10)	0.0884 (19)	0.0001 (9)	0.0288 (12)	0.0013 (11)
O2A	0.0417 (12)	0.0342 (11)	0.0516 (13)	0.0121 (9)	0.0150 (10)	0.0003 (9)
O3A	0.0480 (13)	0.0313 (11)	0.0597 (14)	0.0078 (9)	0.0117 (11)	−0.0068 (10)
N1A	0.0272 (11)	0.0303 (12)	0.0479 (14)	0.0071 (9)	0.0080 (10)	0.0030 (10)
N2A	0.0297 (12)	0.0289 (11)	0.0574 (16)	0.0030 (9)	0.0051 (11)	0.0022 (11)
C1A	0.0356 (15)	0.0258 (13)	0.0509 (17)	0.0055 (11)	0.0165 (13)	0.0037 (12)
C2A	0.0373 (16)	0.0276 (14)	0.081 (3)	0.0062 (12)	0.0234 (16)	0.0005 (15)
C3A	0.050 (2)	0.0388 (17)	0.078 (3)	0.0002 (15)	0.0267 (19)	−0.0095 (17)
C4A	0.0418 (16)	0.0277 (14)	0.0564 (19)	0.0020 (12)	0.0178 (14)	−0.0016 (13)
C5A	0.0382 (15)	0.0262 (13)	0.0571 (19)	0.0022 (11)	0.0176 (14)	0.0046 (13)
C6A	0.0355 (15)	0.0229 (12)	0.0516 (17)	0.0065 (11)	0.0156 (13)	0.0039 (12)
C7A	0.0301 (13)	0.0219 (12)	0.0488 (16)	0.0040 (10)	0.0130 (12)	0.0033 (11)
C8A	0.0350 (14)	0.0259 (12)	0.0399 (15)	0.0045 (11)	0.0103 (12)	0.0022 (11)
C9A	0.0358 (14)	0.0267 (13)	0.0396 (15)	0.0051 (11)	0.0062 (12)	0.0027 (11)
C10A	0.0276 (13)	0.0224 (12)	0.0471 (16)	0.0001 (10)	0.0087 (11)	0.0015 (11)
C11A	0.0314 (13)	0.0221 (12)	0.0429 (15)	−0.0021 (10)	0.0052 (11)	−0.0011 (11)
C12A	0.0335 (14)	0.0285 (13)	0.0470 (16)	−0.0004 (11)	0.0092 (12)	−0.0008 (12)
C13A	0.0420 (16)	0.0352 (15)	0.0526 (18)	0.0009 (13)	0.0172 (14)	−0.0037 (13)
C14A	0.056 (2)	0.0425 (17)	0.0423 (17)	0.0034 (15)	0.0088 (15)	−0.0020 (14)
C15A	0.0499 (19)	0.0361 (15)	0.0445 (17)	0.0011 (14)	−0.0005 (14)	−0.0015 (13)
C16A	0.0347 (15)	0.0252 (13)	0.0494 (17)	−0.0020 (11)	0.0040 (12)	−0.0022 (12)
C17A	0.0302 (13)	0.0223 (12)	0.0560 (18)	0.0012 (10)	0.0132 (12)	0.0021 (12)
C18A	0.0402 (16)	0.0285 (13)	0.0375 (14)	0.0084 (11)	0.0076 (12)	0.0032 (11)
C19A	0.059 (2)	0.0340 (16)	0.059 (2)	0.0102 (15)	0.0152 (17)	−0.0085 (15)
C20A	0.069 (3)	0.0410 (19)	0.083 (3)	0.0050 (18)	0.007 (2)	−0.018 (2)
C21A	0.0352 (15)	0.0387 (16)	0.0541 (19)	0.0032 (13)	0.0027 (13)	−0.0076 (14)
C22A	0.063 (2)	0.0441 (19)	0.077 (3)	0.0123 (17)	0.027 (2)	0.0091 (19)
C23A	0.058 (2)	0.057 (2)	0.076 (3)	−0.0062 (19)	0.017 (2)	−0.016 (2)
Br1B	0.0723 (3)	0.0922 (4)	0.0617 (3)	0.0128 (2)	0.0379 (2)	0.0172 (2)
O1B	0.0324 (10)	0.0288 (9)	0.0510 (12)	−0.0011 (8)	0.0135 (9)	−0.0036 (9)
O2B	0.0337 (11)	0.0357 (11)	0.0547 (13)	−0.0089 (9)	0.0079 (9)	0.0096 (10)
O3B	0.0449 (12)	0.0246 (10)	0.0602 (14)	−0.0025 (9)	0.0186 (10)	0.0083 (9)
N1B	0.0276 (11)	0.0269 (11)	0.0525 (15)	−0.0039 (9)	0.0120 (10)	0.0034 (10)
N2B	0.0397 (14)	0.0270 (11)	0.0487 (15)	−0.0048 (10)	−0.0046 (11)	−0.0049 (11)
C1B	0.0325 (13)	0.0238 (12)	0.0385 (14)	−0.0049 (10)	0.0105 (11)	−0.0016 (11)
C2B	0.0378 (15)	0.0251 (13)	0.065 (2)	−0.0073 (12)	0.0198 (15)	−0.0005 (13)
C3B	0.0513 (18)	0.0253 (13)	0.0535 (18)	−0.0050 (12)	0.0219 (15)	0.0047 (13)
C4B	0.0403 (15)	0.0230 (12)	0.0430 (16)	−0.0018 (11)	0.0130 (13)	0.0011 (11)
C5B	0.0327 (13)	0.0244 (12)	0.0309 (13)	−0.0025 (10)	0.0060 (10)	−0.0011 (10)
C6B	0.0324 (13)	0.0227 (12)	0.0315 (13)	−0.0064 (10)	0.0074 (10)	−0.0018 (10)
C7B	0.0264 (12)	0.0216 (11)	0.0357 (13)	−0.0045 (10)	0.0080 (10)	−0.0014 (10)
C8B	0.0314 (13)	0.0233 (12)	0.0355 (13)	−0.0044 (10)	0.0071 (11)	0.0007 (10)
C9B	0.0317 (14)	0.0232 (12)	0.0411 (15)	−0.0022 (10)	0.0061 (11)	0.0007 (11)
C10B	0.0282 (13)	0.0197 (11)	0.0388 (14)	−0.0009 (9)	0.0045 (11)	−0.0022 (10)
C11B	0.0388 (15)	0.0199 (11)	0.0343 (14)	0.0031 (10)	0.0041 (11)	−0.0008 (10)
C12B	0.0418 (16)	0.0310 (14)	0.0396 (15)	0.0049 (12)	0.0099 (12)	0.0031 (12)
C13B	0.057 (2)	0.0372 (16)	0.0438 (17)	0.0116 (14)	0.0189 (15)	0.0057 (13)
C14B	0.084 (3)	0.0338 (15)	0.0340 (16)	0.0142 (17)	0.0113 (16)	0.0003 (13)
C15B	0.071 (2)	0.0264 (14)	0.0344 (15)	0.0070 (14)	−0.0033 (15)	−0.0045 (12)
C16B	0.0461 (17)	0.0216 (12)	0.0434 (16)	0.0048 (11)	−0.0003 (13)	−0.0036 (11)
C17B	0.0347 (15)	0.0248 (12)	0.0495 (17)	−0.0036 (11)	0.0045 (12)	−0.0049 (12)
C18B	0.0346 (14)	0.0257 (12)	0.0337 (13)	−0.0052 (11)	0.0051 (11)	0.0019 (10)
C19B	0.0531 (19)	0.0238 (13)	0.0491 (18)	−0.0075 (12)	0.0116 (15)	0.0051 (12)
C20B	0.055 (2)	0.0290 (16)	0.104 (4)	−0.0019 (15)	0.011 (2)	0.0128 (19)
C21B	0.0295 (15)	0.0347 (15)	0.071 (2)	−0.0013 (12)	0.0016 (14)	0.0097 (15)
C22B	0.0519 (19)	0.0329 (15)	0.0503 (18)	−0.0120 (13)	0.0150 (15)	−0.0102 (13)
C23B	0.0540 (19)	0.0320 (15)	0.057 (2)	0.0067 (14)	0.0151 (16)	0.0089 (14)

### Geometric parameters (Å, º)

**Table:**

Br1A—C13A	1.906 (3)	Br1B—C13B	1.903 (4)
O1A—C5A	1.225 (4)	O1B—C5B	1.232 (4)
O2A—C18A	1.220 (4)	O2B—C18B	1.214 (4)
O3A—C18A	1.332 (4)	O3B—C18B	1.338 (4)
O3A—C19A	1.447 (4)	O3B—C19B	1.457 (3)
N1A—C1A	1.374 (4)	N1B—C1B	1.371 (4)
N1A—C9A	1.395 (4)	N1B—C9B	1.393 (4)
N1A—H1AA	0.8800	N1B—H1BA	0.8800
N2A—C17A	1.362 (5)	N2B—C16B	1.365 (5)
N2A—C16A	1.367 (4)	N2B—C17B	1.374 (4)
N2A—H2AC	0.8800	N2B—H2BC	0.8800
C1A—C6A	1.356 (4)	C1B—C6B	1.353 (4)
C1A—C2A	1.509 (4)	C1B—C2B	1.503 (4)
C2A—C3A	1.556 (5)	C2B—C3B	1.528 (5)
C2A—H2AA	0.9900	C2B—H2BA	0.9900
C2A—H2AB	0.9900	C2B—H2BB	0.9900
C3A—C4A	1.515 (5)	C3B—C4B	1.536 (4)
C3A—H3AA	0.9900	C3B—H3BA	0.9900
C3A—H3AB	0.9900	C3B—H3BB	0.9900
C4A—C22A	1.524 (5)	C4B—C23B	1.526 (4)
C4A—C23A	1.538 (5)	C4B—C22B	1.535 (5)
C4A—C5A	1.539 (4)	C4B—C5B	1.536 (4)
C5A—C6A	1.457 (4)	C5B—C6B	1.450 (4)
C6A—C7A	1.516 (4)	C6B—C7B	1.516 (3)
C7A—C10A	1.512 (4)	C7B—C10B	1.508 (4)
C7A—C8A	1.521 (4)	C7B—C8B	1.523 (4)
C7A—H7AA	1.0000	C7B—H7BA	1.0000
C8A—C9A	1.356 (4)	C8B—C9B	1.360 (4)
C8A—C18A	1.472 (4)	C8B—C18B	1.468 (4)
C9A—C21A	1.495 (4)	C9B—C21B	1.500 (4)
C10A—C17A	1.371 (4)	C10B—C17B	1.371 (4)
C10A—C11A	1.437 (4)	C10B—C11B	1.446 (4)
C11A—C12A	1.405 (4)	C11B—C12B	1.403 (4)
C11A—C16A	1.419 (4)	C11B—C16B	1.424 (4)
C12A—C13A	1.371 (5)	C12B—C13B	1.379 (5)
C12A—H12A	0.9500	C12B—H12B	0.9500
C13A—C14A	1.406 (5)	C13B—C14B	1.406 (6)
C14A—C15A	1.380 (5)	C14B—C15B	1.369 (6)
C14A—H14A	0.9500	C14B—H14B	0.9500
C15A—C16A	1.389 (5)	C15B—C16B	1.397 (5)
C15A—H15A	0.9500	C15B—H15B	0.9500
C17A—H17A	0.9500	C17B—H17B	0.9500
C19A—C20A	1.480 (6)	C19B—C20B	1.491 (5)
C19A—H19A	0.9900	C19B—H19C	0.9900
C19A—H19B	0.9900	C19B—H19D	0.9900
C20A—H20A	0.9800	C20B—H20D	0.9800
C20A—H20B	0.9800	C20B—H20E	0.9800
C20A—H20C	0.9800	C20B—H20F	0.9800
C21A—H21A	0.9800	C21B—H21D	0.9800
C21A—H21B	0.9800	C21B—H21E	0.9800
C21A—H21C	0.9800	C21B—H21F	0.9800
C22A—H22A	0.9800	C22B—H22D	0.9800
C22A—H22B	0.9800	C22B—H22E	0.9800
C22A—H22C	0.9800	C22B—H22F	0.9800
C23A—H23A	0.9800	C23B—H23G	0.9800
C23A—H23B	0.9800	C23B—H23D	0.9800
C23A—H23C	0.9800	C23B—H23E	0.9800
			
C18A—O3A—C19A	116.9 (3)	C18B—O3B—C19B	117.1 (2)
C1A—N1A—C9A	122.6 (3)	C1B—N1B—C9B	122.1 (2)
C1A—N1A—H1AA	118.7	C1B—N1B—H1BA	118.9
C9A—N1A—H1AA	118.7	C9B—N1B—H1BA	118.9
C17A—N2A—C16A	109.0 (3)	C16B—N2B—C17B	108.5 (3)
C17A—N2A—H2AC	125.5	C16B—N2B—H2BC	125.8
C16A—N2A—H2AC	125.5	C17B—N2B—H2BC	125.8
C6A—C1A—N1A	120.6 (3)	C6B—C1B—N1B	120.6 (2)
C6A—C1A—C2A	123.1 (3)	C6B—C1B—C2B	124.1 (3)
N1A—C1A—C2A	116.2 (3)	N1B—C1B—C2B	115.2 (2)
C1A—C2A—C3A	109.6 (3)	C1B—C2B—C3B	110.0 (3)
C1A—C2A—H2AA	109.8	C1B—C2B—H2BA	109.7
C3A—C2A—H2AA	109.8	C3B—C2B—H2BA	109.7
C1A—C2A—H2AB	109.8	C1B—C2B—H2BB	109.7
C3A—C2A—H2AB	109.8	C3B—C2B—H2BB	109.7
H2AA—C2A—H2AB	108.2	H2BA—C2B—H2BB	108.2
C4A—C3A—C2A	112.4 (3)	C2B—C3B—C4B	112.8 (3)
C4A—C3A—H3AA	109.1	C2B—C3B—H3BA	109.0
C2A—C3A—H3AA	109.1	C4B—C3B—H3BA	109.0
C4A—C3A—H3AB	109.1	C2B—C3B—H3BB	109.0
C2A—C3A—H3AB	109.1	C4B—C3B—H3BB	109.0
H3AA—C3A—H3AB	107.9	H3BA—C3B—H3BB	107.8
C3A—C4A—C22A	110.9 (3)	C23B—C4B—C22B	110.2 (3)
C3A—C4A—C23A	109.8 (3)	C23B—C4B—C5B	109.4 (3)
C22A—C4A—C23A	108.6 (3)	C22B—C4B—C5B	107.1 (2)
C3A—C4A—C5A	111.2 (3)	C23B—C4B—C3B	109.2 (3)
C22A—C4A—C5A	106.2 (3)	C22B—C4B—C3B	110.5 (3)
C23A—C4A—C5A	110.1 (3)	C5B—C4B—C3B	110.4 (2)
O1A—C5A—C6A	120.3 (3)	O1B—C5B—C6B	120.8 (2)
O1A—C5A—C4A	120.2 (3)	O1B—C5B—C4B	119.6 (2)
C6A—C5A—C4A	119.5 (3)	C6B—C5B—C4B	119.6 (2)
C1A—C6A—C5A	121.0 (3)	C1B—C6B—C5B	120.1 (2)
C1A—C6A—C7A	120.5 (3)	C1B—C6B—C7B	120.5 (2)
C5A—C6A—C7A	118.4 (3)	C5B—C6B—C7B	119.3 (2)
C10A—C7A—C6A	109.8 (3)	C10B—C7B—C6B	111.7 (2)
C10A—C7A—C8A	112.4 (2)	C10B—C7B—C8B	111.9 (2)
C6A—C7A—C8A	111.1 (2)	C6B—C7B—C8B	110.5 (2)
C10A—C7A—H7AA	107.8	C10B—C7B—H7BA	107.5
C6A—C7A—H7AA	107.8	C6B—C7B—H7BA	107.5
C8A—C7A—H7AA	107.8	C8B—C7B—H7BA	107.5
C9A—C8A—C18A	124.4 (3)	C9B—C8B—C18B	124.6 (3)
C9A—C8A—C7A	121.5 (3)	C9B—C8B—C7B	121.1 (2)
C18A—C8A—C7A	114.0 (2)	C18B—C8B—C7B	114.3 (2)
C8A—C9A—N1A	119.2 (3)	C8B—C9B—N1B	119.2 (3)
C8A—C9A—C21A	127.7 (3)	C8B—C9B—C21B	128.7 (3)
N1A—C9A—C21A	113.0 (3)	N1B—C9B—C21B	112.1 (2)
C17A—C10A—C11A	105.9 (3)	C17B—C10B—C11B	105.7 (3)
C17A—C10A—C7A	126.1 (3)	C17B—C10B—C7B	124.9 (3)
C11A—C10A—C7A	127.9 (3)	C11B—C10B—C7B	129.4 (2)
C12A—C11A—C16A	118.4 (3)	C12B—C11B—C16B	118.7 (3)
C12A—C11A—C10A	134.9 (3)	C12B—C11B—C10B	134.8 (3)
C16A—C11A—C10A	106.8 (3)	C16B—C11B—C10B	106.5 (3)
C13A—C12A—C11A	118.3 (3)	C13B—C12B—C11B	118.0 (3)
C13A—C12A—H12A	120.8	C13B—C12B—H12B	121.0
C11A—C12A—H12A	120.8	C11B—C12B—H12B	121.0
C12A—C13A—C14A	123.0 (3)	C12B—C13B—C14B	123.0 (3)
C12A—C13A—Br1A	118.5 (3)	C12B—C13B—Br1B	119.1 (3)
C14A—C13A—Br1A	118.5 (3)	C14B—C13B—Br1B	117.9 (3)
C15A—C14A—C13A	119.6 (3)	C15B—C14B—C13B	119.9 (3)
C15A—C14A—H14A	120.2	C15B—C14B—H14B	120.0
C13A—C14A—H14A	120.2	C13B—C14B—H14B	120.0
C14A—C15A—C16A	118.1 (3)	C14B—C15B—C16B	118.3 (3)
C14A—C15A—H15A	120.9	C14B—C15B—H15B	120.9
C16A—C15A—H15A	120.9	C16B—C15B—H15B	120.9
N2A—C16A—C15A	129.8 (3)	N2B—C16B—C15B	129.7 (3)
N2A—C16A—C11A	107.6 (3)	N2B—C16B—C11B	108.2 (3)
C15A—C16A—C11A	122.6 (3)	C15B—C16B—C11B	122.1 (3)
N2A—C17A—C10A	110.7 (3)	C10B—C17B—N2B	111.1 (3)
N2A—C17A—H17A	124.6	C10B—C17B—H17B	124.4
C10A—C17A—H17A	124.6	N2B—C17B—H17B	124.4
O2A—C18A—O3A	122.6 (3)	O2B—C18B—O3B	121.9 (3)
O2A—C18A—C8A	122.4 (3)	O2B—C18B—C8B	122.7 (3)
O3A—C18A—C8A	115.0 (3)	O3B—C18B—C8B	115.3 (2)
O3A—C19A—C20A	106.7 (3)	O3B—C19B—C20B	106.0 (3)
O3A—C19A—H19A	110.4	O3B—C19B—H19C	110.5
C20A—C19A—H19A	110.4	C20B—C19B—H19C	110.5
O3A—C19A—H19B	110.4	O3B—C19B—H19D	110.5
C20A—C19A—H19B	110.4	C20B—C19B—H19D	110.5
H19A—C19A—H19B	108.6	H19C—C19B—H19D	108.7
C19A—C20A—H20A	109.5	C19B—C20B—H20D	109.5
C19A—C20A—H20B	109.5	C19B—C20B—H20E	109.5
H20A—C20A—H20B	109.5	H20D—C20B—H20E	109.5
C19A—C20A—H20C	109.5	C19B—C20B—H20F	109.5
H20A—C20A—H20C	109.5	H20D—C20B—H20F	109.5
H20B—C20A—H20C	109.5	H20E—C20B—H20F	109.5
C9A—C21A—H21A	109.5	C9B—C21B—H21D	109.5
C9A—C21A—H21B	109.5	C9B—C21B—H21E	109.5
H21A—C21A—H21B	109.5	H21D—C21B—H21E	109.5
C9A—C21A—H21C	109.5	C9B—C21B—H21F	109.5
H21A—C21A—H21C	109.5	H21D—C21B—H21F	109.5
H21B—C21A—H21C	109.5	H21E—C21B—H21F	109.5
C4A—C22A—H22A	109.5	C4B—C22B—H22D	109.5
C4A—C22A—H22B	109.5	C4B—C22B—H22E	109.5
H22A—C22A—H22B	109.5	H22D—C22B—H22E	109.5
C4A—C22A—H22C	109.5	C4B—C22B—H22F	109.5
H22A—C22A—H22C	109.5	H22D—C22B—H22F	109.5
H22B—C22A—H22C	109.5	H22E—C22B—H22F	109.5
C4A—C23A—H23A	109.5	C4B—C23B—H23G	109.5
C4A—C23A—H23B	109.5	C4B—C23B—H23D	109.5
H23A—C23A—H23B	109.5	H23G—C23B—H23D	109.5
C4A—C23A—H23C	109.5	C4B—C23B—H23E	109.5
H23A—C23A—H23C	109.5	H23G—C23B—H23E	109.5
H23B—C23A—H23C	109.5	H23D—C23B—H23E	109.5
			
C9A—N1A—C1A—C6A	9.0 (5)	C9B—N1B—C1B—C6B	−12.8 (5)
C9A—N1A—C1A—C2A	−171.1 (3)	C9B—N1B—C1B—C2B	166.0 (3)
C6A—C1A—C2A—C3A	25.3 (5)	C6B—C1B—C2B—C3B	−19.8 (4)
N1A—C1A—C2A—C3A	−154.6 (3)	N1B—C1B—C2B—C3B	161.5 (3)
C1A—C2A—C3A—C4A	−52.8 (4)	C1B—C2B—C3B—C4B	50.6 (4)
C2A—C3A—C4A—C22A	−67.6 (4)	C2B—C3B—C4B—C23B	−171.3 (3)
C2A—C3A—C4A—C23A	172.4 (3)	C2B—C3B—C4B—C22B	67.4 (3)
C2A—C3A—C4A—C5A	50.3 (4)	C2B—C3B—C4B—C5B	−50.9 (4)
C3A—C4A—C5A—O1A	160.1 (4)	C23B—C4B—C5B—O1B	−42.1 (4)
C22A—C4A—C5A—O1A	−79.2 (4)	C22B—C4B—C5B—O1B	77.3 (3)
C23A—C4A—C5A—O1A	38.2 (5)	C3B—C4B—C5B—O1B	−162.4 (3)
C3A—C4A—C5A—C6A	−20.3 (5)	C23B—C4B—C5B—C6B	140.8 (3)
C22A—C4A—C5A—C6A	100.4 (4)	C22B—C4B—C5B—C6B	−99.8 (3)
C23A—C4A—C5A—C6A	−142.3 (3)	C3B—C4B—C5B—C6B	20.6 (4)
N1A—C1A—C6A—C5A	−175.4 (3)	N1B—C1B—C6B—C5B	167.9 (3)
C2A—C1A—C6A—C5A	4.7 (5)	C2B—C1B—C6B—C5B	−10.7 (5)
N1A—C1A—C6A—C7A	8.6 (5)	N1B—C1B—C6B—C7B	−8.2 (4)
C2A—C1A—C6A—C7A	−171.3 (3)	C2B—C1B—C6B—C7B	173.1 (3)
O1A—C5A—C6A—C1A	171.8 (3)	O1B—C5B—C6B—C1B	−166.9 (3)
C4A—C5A—C6A—C1A	−7.8 (5)	C4B—C5B—C6B—C1B	10.1 (4)
O1A—C5A—C6A—C7A	−12.2 (5)	O1B—C5B—C6B—C7B	9.3 (4)
C4A—C5A—C6A—C7A	168.2 (3)	C4B—C5B—C6B—C7B	−173.7 (2)
C1A—C6A—C7A—C10A	103.4 (3)	C1B—C6B—C7B—C10B	−101.1 (3)
C5A—C6A—C7A—C10A	−72.6 (4)	C5B—C6B—C7B—C10B	82.7 (3)
C1A—C6A—C7A—C8A	−21.5 (4)	C1B—C6B—C7B—C8B	24.2 (4)
C5A—C6A—C7A—C8A	162.4 (3)	C5B—C6B—C7B—C8B	−152.0 (2)
C10A—C7A—C8A—C9A	−103.7 (3)	C10B—C7B—C8B—C9B	102.7 (3)
C6A—C7A—C8A—C9A	19.7 (4)	C6B—C7B—C8B—C9B	−22.5 (4)
C10A—C7A—C8A—C18A	74.4 (3)	C10B—C7B—C8B—C18B	−77.3 (3)
C6A—C7A—C8A—C18A	−162.2 (3)	C6B—C7B—C8B—C18B	157.5 (2)
C18A—C8A—C9A—N1A	177.3 (3)	C18B—C8B—C9B—N1B	−175.1 (3)
C7A—C8A—C9A—N1A	−4.9 (4)	C7B—C8B—C9B—N1B	4.8 (4)
C18A—C8A—C9A—C21A	−0.2 (5)	C18B—C8B—C9B—C21B	2.4 (5)
C7A—C8A—C9A—C21A	177.7 (3)	C7B—C8B—C9B—C21B	−177.6 (3)
C1A—N1A—C9A—C8A	−10.9 (5)	C1B—N1B—C9B—C8B	14.5 (5)
C1A—N1A—C9A—C21A	166.9 (3)	C1B—N1B—C9B—C21B	−163.5 (3)
C6A—C7A—C10A—C17A	132.8 (3)	C6B—C7B—C10B—C17B	−127.5 (3)
C8A—C7A—C10A—C17A	−102.9 (3)	C8B—C7B—C10B—C17B	108.0 (3)
C6A—C7A—C10A—C11A	−42.2 (4)	C6B—C7B—C10B—C11B	52.7 (4)
C8A—C7A—C10A—C11A	82.1 (3)	C8B—C7B—C10B—C11B	−71.8 (3)
C17A—C10A—C11A—C12A	178.0 (3)	C17B—C10B—C11B—C12B	−177.8 (3)
C7A—C10A—C11A—C12A	−6.2 (5)	C7B—C10B—C11B—C12B	1.9 (5)
C17A—C10A—C11A—C16A	−1.0 (3)	C17B—C10B—C11B—C16B	0.7 (3)
C7A—C10A—C11A—C16A	174.8 (3)	C7B—C10B—C11B—C16B	−179.5 (3)
C16A—C11A—C12A—C13A	−1.6 (4)	C16B—C11B—C12B—C13B	1.2 (4)
C10A—C11A—C12A—C13A	179.5 (3)	C10B—C11B—C12B—C13B	179.7 (3)
C11A—C12A—C13A—C14A	0.3 (5)	C11B—C12B—C13B—C14B	0.8 (5)
C11A—C12A—C13A—Br1A	−179.6 (2)	C11B—C12B—C13B—Br1B	179.5 (2)
C12A—C13A—C14A—C15A	1.3 (5)	C12B—C13B—C14B—C15B	−1.5 (5)
Br1A—C13A—C14A—C15A	−178.8 (3)	Br1B—C13B—C14B—C15B	179.8 (2)
C13A—C14A—C15A—C16A	−1.5 (5)	C13B—C14B—C15B—C16B	0.0 (5)
C17A—N2A—C16A—C15A	179.6 (3)	C17B—N2B—C16B—C15B	−178.7 (3)
C17A—N2A—C16A—C11A	−1.4 (3)	C17B—N2B—C16B—C11B	0.4 (3)
C14A—C15A—C16A—N2A	179.1 (3)	C14B—C15B—C16B—N2B	−179.0 (3)
C14A—C15A—C16A—C11A	0.2 (5)	C14B—C15B—C16B—C11B	2.0 (4)
C12A—C11A—C16A—N2A	−177.7 (2)	C12B—C11B—C16B—N2B	178.1 (2)
C10A—C11A—C16A—N2A	1.5 (3)	C10B—C11B—C16B—N2B	−0.7 (3)
C12A—C11A—C16A—C15A	1.4 (4)	C12B—C11B—C16B—C15B	−2.7 (4)
C10A—C11A—C16A—C15A	−179.4 (3)	C10B—C11B—C16B—C15B	178.5 (3)
C16A—N2A—C17A—C10A	0.8 (3)	C11B—C10B—C17B—N2B	−0.5 (3)
C11A—C10A—C17A—N2A	0.1 (3)	C7B—C10B—C17B—N2B	179.7 (2)
C7A—C10A—C17A—N2A	−175.8 (3)	C16B—N2B—C17B—C10B	0.1 (3)
C19A—O3A—C18A—O2A	−0.1 (5)	C19B—O3B—C18B—O2B	1.6 (4)
C19A—O3A—C18A—C8A	178.3 (3)	C19B—O3B—C18B—C8B	−178.7 (3)
C9A—C8A—C18A—O2A	−171.6 (3)	C9B—C8B—C18B—O2B	170.5 (3)
C7A—C8A—C18A—O2A	10.4 (4)	C7B—C8B—C18B—O2B	−9.5 (4)
C9A—C8A—C18A—O3A	10.0 (4)	C9B—C8B—C18B—O3B	−9.3 (4)
C7A—C8A—C18A—O3A	−168.1 (3)	C7B—C8B—C18B—O3B	170.7 (2)
C18A—O3A—C19A—C20A	−178.3 (3)	C18B—O3B—C19B—C20B	−176.5 (3)

### Hydrogen-bond geometry (Å, º)

**Table:**

*D*—H···*A*	*D*—H	H···*A*	*D*···*A*	*D*—H···*A*
C2*A*—H2*AB*···O1*B*	0.99	2.51	3.296 (4)	136
C21*B*—H21*D*···O3*B*	0.98	2.17	2.749 (4)	116
N1*A*—H1*AA*···O1*B*	0.88	2.01	2.870 (3)	165
N2*A*—H2*AC*···O2*B*^i^	0.88	1.94	2.807 (3)	167
N1*B*—H1*BA*···O1*A*^ii^	0.88	1.97	2.845 (3)	173
N2*B*—H2*BC*···O2*A*^iii^	0.88	2.05	2.889 (3)	159

Symmetry codes: (i) −*x*, *y*+1/2, −*z*+1/2; (ii) *x*+1, *y*, *z*; (iii) −*x*, *y*−1/2, −*z*+1/2.
